# Features of Myocarditis: Morphological Differential Diagnosis in Post-COVID-19 Children

**DOI:** 10.3390/diagnostics13152499

**Published:** 2023-07-27

**Authors:** Vadim Karev, Anastasia Ya Starshinova, Anzhela Glushkova, Dmitry Kudlay, Anna Starshinova

**Affiliations:** 1 Children’s Clinical Research Center for Infectious Diseases, St. Petersburg 194100, Russia; vadimkarev@yandex.ru; 2 Almazov National Medical Research Centre, St. Petersburg 197341, Russia; 3 Pediatric Faculty, State Pediatric Medical University, St. Petersburg 194100, Russia; asya.starshinova@mail.ru; 4 Medical Faculty, Pavlov First Saint Petersburg State Medical University, St. Petersburg 197022, Russia; angela_glushkova@yahoo.com; 5 Department of Pharmacology, Institute of Pharmacy, I.M. Sechenov First Moscow State Medical University, Moscow 119991, Russia; d624254@gmail.com; 6 Institute of Immunology FMBA of Russia, Moscow 115478, Russia

**Keywords:** SARS-CoV-2 infection, immunohistochemical and ultrastructural myocardial studies, heart transplantation, virus transmission

## Abstract

Myocarditis is characterized by dysfunction and destruction of cardiomyocytes, infiltrative inflammation, and development of fibrosis. Late diagnosis of myocarditis has been a serious global health problem, especially due to the spread of a new coronavirus infection. The aim of this review is to identify differences between myocarditis of viral etiology, including SARS-CoV-2 lesions, based on instrumental and pathomorphological findings. Material and Methods: We analyzed publications covering the period from December 2019 to May 2023, published in publicly accessible international databases (“Medline”, “PubMed”, “Scopus”), with queries for the keywords “myocarditis”, “children”, “cardiovascular inflammation”, “COVID-19”, “SARS-CoV-2”, “severe acute respiratory syndrome coronavirus 2”, “differential diagnosis”. Results: It was found that no unambiguous morphological criteria for the diagnosis of myocarditis coupled to SARS-CoV-2 lesions were identified. However, the detected histopathological changes such as virus-associated degeneration, apoptosis, cardiomyocyte necrosis, moderate interstitial hyperemia, myocardial tissue oedema, and capillary endothelial cell dysfunction were the major markers of SARS-CoV-2 infection. Conclusion: It is necessary further reconsider morphological criteria to diagnose SARS-CoV-2-caused myocarditis, rather than solely relying on detecting viral RNA by PCR as the sole evidence-based criterion. Similar issues accompany diagnostics of myocardial lesions associated with other viral infections. Evidence for an etiological diagnosis of myocarditis can be provided by a comprehensive analysis of the diagnostic criteria obtained, confirming virus exposure, followed by development of distinct clinical symptoms, MRI and CT changes, and morphological criteria.

## 1. Introduction

In March 2020, the world was confronted with a novel infectious pathogen that causes a new coronavirus infection: COVID-19 [1,2].

The rapid spread of the SARS-CoV-2 virus led to the World Health Organization (WHO) declaring a pandemic in March 2020 [3]. Since the emergence of COVID-19, according to WHO monitoring, more than 766 million confirmed cases have been reported, among which, about 6 million patients have died and more than 13 million people were vaccinated [4]. Around 8.5% of all COVID-19 cases were found in children [5]; most had it in a mild or asymptomatic form with low mortality [6,7]. However, critical conditions requiring intensive care and deaths have also been reported in pediatric practice [8,9]. It is clear that a decline in COVID-19 pandemic tension has been observed, but investigating the effects related to the infection and sequelae remain far from being fully elucidated [10,11,12].

The main pathological changes and long-term consequences may be associated with cardiovascular damage in COVID-19 patients due to arising systemic inflammatory response, developing myocardial ischemia and vasculitis, often coupled to autoimmune inflammation [13,14,15].

It has been currently understood that SARS-CoV-2 results in hypoperfusion, overstimulation of β-adrenergic receptors, thrombosis, and thromboembolism, triggering myocardial damage, with unknown long-term consequences [15,16].

In pediatric practice, myocardial lesions pose a threat in any viral infection [9,17]. The lack of prominent and specific changes emerging, either as clinical symptoms or instrumental signs, leads to a need to perform myocardial biopsy for pathomorphological examination [11,18,19].

Myocarditis is a condition caused by acute or chronic inflammation of cardiomyocytes, subsequently resulting in myocardial edema, damage, and necrosis [20]. Myocarditis is characterized by cardiomyocyte dysfunction and death, infiltrative inflammation, and development of fibrosis. Late diagnosis of myocarditis has been recognized as a serious global health problem, especially when coupled with the spread of a novel coronavirus infection [21].

COVID-19 is one of the major causes associated with high pediatric morbidity and mortality [17]. The exact proportion of verified myocarditis in children remains currently unknown due to often mild or asymptomatic cases, coupled with minimal and non-specific symptoms [22,23]. However, it is known that, in 12% of cases, myocarditis was the cause of death in adolescents and young people who died from sudden death syndrome [24].

Myocarditis-related clinical manifestations can vary widely, from minimal symptoms to severe conditions, such as chronic congestive heart failure, dilated cardiomyopathy, refractory arrhythmias, and cardiogenic shock, collectively resulting in heart transplantation or sudden cardiac death [24,25,26]. Prolonged asymptomatic myocardial inflammation leads to emerging heart failure and chronic dilated cardiomyopathy, serving as an indication for heart transplantation in children older than one year of age [27,28].

Here, we aimed to identify potential differences between myocarditis of viral etiology, including that caused by SARS-CoV-2, by assessing instrumental and pathomorphological findings.

## 2. Material and Methods

We analyzed publications within the period December 2019 to May 2023, published in openly accessible international databases (“Medline”, “PubMed”, “Scopus”), with queries for the keywords “myocarditis”, “children”, “cardiovascular inflammation”, “COVID-19”, “SARS-CoV-2”, “severe acute respiratory syndrome coronavirus 2”, and “differential diagnosis”. Morphologic diagnosis of myocarditis was performed with the use of updated WHO criteria, adopted in 2013 by the European Society of Cardiology [29].

## 3. Results

The morphological examination is an important step in the diagnosis and confirmation of disease etiology. To visualize expression of viral antigens in various histological cardiac sites using immunohistochemistry method, monoclonal or polyclonal antibodies were used.

Despite advances in myocardial imaging, direct tissue examination remains the reference standard for myocarditis diagnostics. The 1987 Dallas criteria provide a standard histological basis to diagnose myocarditis based on detection of inflammatory cell infiltrates and myocyte necrosis that cannot be interpreted by coronary artery disease or other pathogenesis [29]. However, due to the focal nature of myocardial infiltrates and random sampling, a high false negative rate has been reported [30].

Immunohistochemistry increased the sensitivity of detecting inflammatory cellular infiltrates that can also reveal upregulated expression of HLA and pro-inflammatory cellular markers to confirm myocarditis diagnosis, in case it is not verified by routine histology [31,32].

The major mechanisms for myocardial damage in COVID-19 patients result from a cytokine storm due to unbalanced response of T-helper 1 (TH1 cells) and T-helper 2 (TH2 cells) [33]; respiratory dysfunction and hypoxemia; and decreased ACE2 activity, exerting a protective effect on the cardiovascular system as an arm for counter-regulating angiotensin II signaling [34].

Some studies have demonstrated that SARS-CoV-2-caused myocardial injury may be related to increased viscosity, enhanced coagulation cascade, pro-inflammatory effects, and endothelial cell dysfunction [35]. Moreover, it was also associated with cardiomyocyte degeneration, hypertrophy and necrosis, moderate interstitial hyperemia, and edema, along with infiltration by lymphocytes, monocytes, and neutrophils, but lacking detected viral markers in the myocardial tissue [36,37]. In addition, a myocardial injury may include atherosclerotic plaque rupture, coronary vasospasm, hypoxic vascular injury, and direct endothelial or microthrombogenesis [37]. Pericytes infected with the SARS-CoV-2 virus were shown to elicit dysfunction of capillary endothelial cells or microvessels, followed by related necrosis [36].

It was noted that acute coronary syndrome may be one of the initial manifestations during COVID-19 infection, being able to range from ST-elevation myocardial infarction to takotsubo cardiomyopathy, whereas ischemia and myocardial infarction may be secondary to plaque rupture due to a stress response or thrombosis [38]. In addition, it was found that SARS-CoV-2 may be detected in macrophages within cardiac tissue, allowing to enter it during transient viremia [39]; as well, viral particles were observed in endothelial cells along with accumulated myocardial inflammatory cells and signs of endothelial and inflammatory cell death [40].

Considering the pantropy of diverse viruses, expression of the relevant antigens may be observed in cardiomyocytes, vascular endothelial cells, or endocardium, as well as in inflammatory infiltrate cells, mesothelial cells of the pericardium, or in the structures of the cardiac conduction system [41]. Detection of expressed antigens related to cardiotropic viruses in various cardiac structures, on the one hand, contributes to verifying etiology of heart damage, and, on the other hand, may markedly expand understanding of the underlying pathogenesis behind virus-induced heart diseases. The undoubted advantage of the immunohistochemistry approach is that it allows one to perform studies retrospectively by using histology samples obtained from archival paraffin blocks.

**Figure 1:**
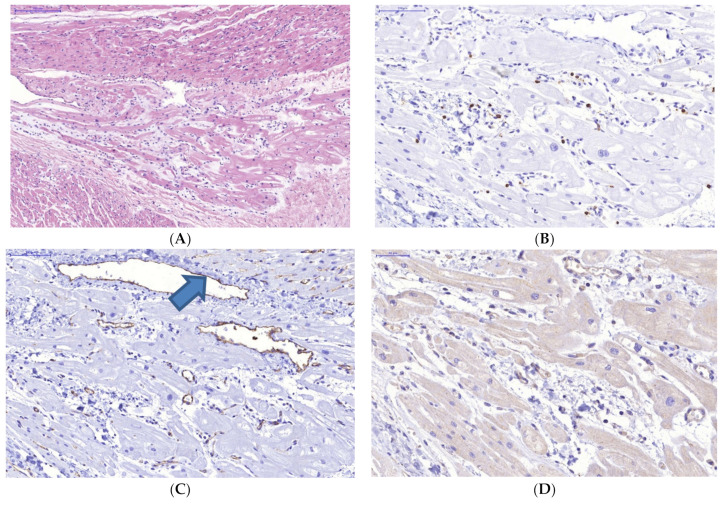
Focal acute polymorphic cell myocarditis (**A**,**B**), pathological alterations in intramural blood vessels (**C**), and expression of SARS-CoV-2 spike antigen (**D**) by endothelial and smooth muscle cells of myocardial blood vessels. A—H&E, B—IHH, mouse monoclonal anti-CD45-specific staining (Thermo, Waltham, MA, USA); C—IHH, mouse monoclonal anti-CD31-specific staining (Thermo, Waltham, MA, USA); D—IHH, rabbit polyclonal anti-SARS-CoV-2 Spike-specific staining (GeneTex, Irvine, CA, USA), DAB. Scale range: segment (**A**)—2000 μm, (**B**,**C**)—100 μm; (**D**)—500 µm.

In the heart, along with cardiomyocyte dystrophic changes of varying severity, manifestations of circulatory disorders, and formation of unevenly evident intermuscular edema, there are also small foci of polymorphocellular interstitial “aggressive” infiltration (of lymphohistiocytic nature, bearing 10–15 infiltrating cells observed in the field of view at microscope magnification of ×400) featured with invasion of single lymphocytes within the muscle fibers, showing signs of damage (Figure 1A,B).

Outside the foci of exudative inflammation, smooth muscle and endothelial cells within arterial-type intramural blood vessels expressed SARS-CoV-2 Spike protein (Figure 1D).

The differential diagnosis for myocardial damage is carried out by diverse assessments, in case no unequivocal data confirming SARS-CoV-2-infection are available. Commercial antibodies specific to antigens of herpes simplex virus types 1 and 2, herpes simplex virus type 6, cytomegalovirus, Epstein–Barr virus, varicella-zoster, enterovirus, and parvovirus B19 are among the most widely used for differential diagnostics.

**Figure 2:**
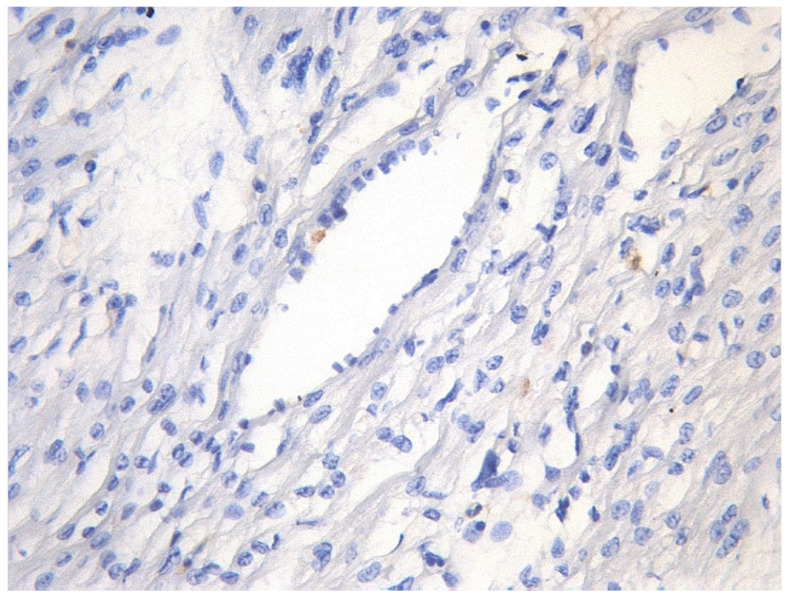
Expression of antigens of herpes simplex virus type 2 (brown staining) in non-parenchymal myocardial cells in generalized intrauterine herpes virus infection. IHH, rabbit polyclonal anti-HSV-2 (BioGenex, Fremont, CA, USA), DAB. SW. ×200.

During generalized viral infection, herpes simplex viruses (the Herpesviridae family) often infect epithelial and endothelial cells, fibroblasts, smooth muscle cells, nerve cells, and macrophages, altering their antigenic and functional properties, as well as stimulating host immune response [41]. Such viruses in myocarditis are more often detected in fibroblasts and vascular endotheliocytes, with fewer found in cardiomyocytes (Figure 2).

Herpes simplex viruses enter the heart through the atrial septum, which is specifically enriched in sensory nerve endings and fibers as well as ganglia [42,43]. They can be localized either in the atria or ventricles, evidencing that this herpes simplex virus results in in situ isolated lesion while infection spreads along the nerve fibers. Morphological changes are present as increased size of the cell nucleus, peripherally positioned chromatin with its further disappearance (intranuclear inclusions), fragmentation of nucleoli, formation of multiple cytoplasmic polysomes, and elevated macromolecule permeability [44]. The transformation of affected cells often results in giant cell metamorphosis. Herpesvirus infection is often accompanied by emerging necrosis coupled to dusty or clumpy nuclear rexis of dead cells and scanty perifocal polymorphonuclear leukocyte infiltration.

**Figure 3:**
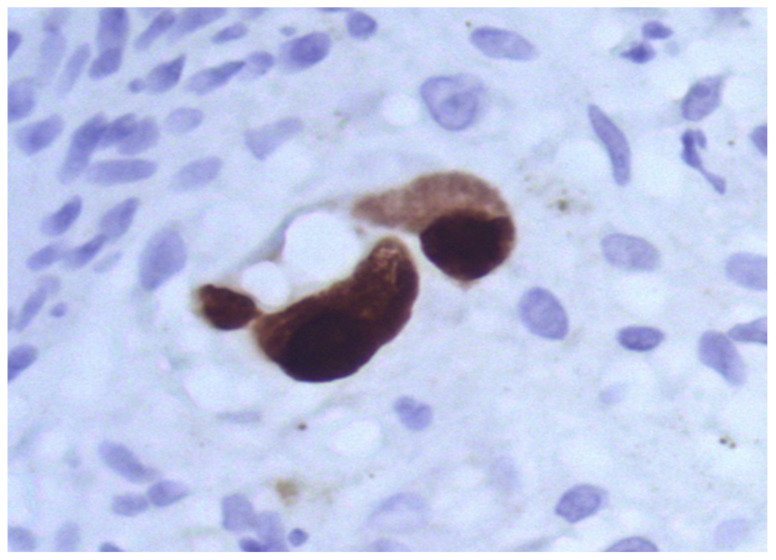
Expression of cytomegalovirus antigens (brown staining) in transformed endothelial cells during generalized intrauterine cytomegalovirus infection. IHH, mouse monoclonal anti-CMV-specific staining (DAKO, Santa Clara, CA, USA), DAB. SW. ×400.

In parenchymal and non-parenchymal cells, herpes simplex virus antigens are expressed in the nuclei and/or cytoplasm. Similar to other herpes family viruses, cytomegalovirus displays a prominent pantropic behavior; however, cytomegalovirus-related damage to cardiomyocytes followed by relevant giant cell transformation occurs rarely, usually due to severe immunodeficiency [45]. Most often, cytomegalovirus-triggered heart lesions are limited to virus-induced alterations in the vascular endothelium (Figure 3) and parietal and valvular endocardium, as well as expression of virus-specific antigens observed in interstitial inflammatory infiltration and endothelial cells [46].

The early cytomegalovirus antigen is expressed mainly in infected cell (parenchymal cells, endotheliocytes, leukocytes, macrophages) nucleus, whereas its late antigen is found in the nucleus and cytoplasm. It should be emphasized that infected parenchymal or non-parenchymal cells expressing cytomegalovirus antigens do not always undergo a giant cell transformation, which can lead to related morphological underdiagnosis of organ lesions.

After long-lasting persistence, herpes virus type 6 can elicit myocarditis. More often, focal myocarditis is described in the basal parts of the interventricular septum, being accompanied by rhythm disturbances, particularly, blockade of electrical impulse conduction [47]. In myocarditis, virus antigens are expressed in cardiomyocytes, vascular endothelial cells, and inflammatory infiltrate cells [48]. The major pathogenic effect related to herpes virus type 6 on the myocardium is exerted indirectly via endothelial cell damage.

**Figure 4:**
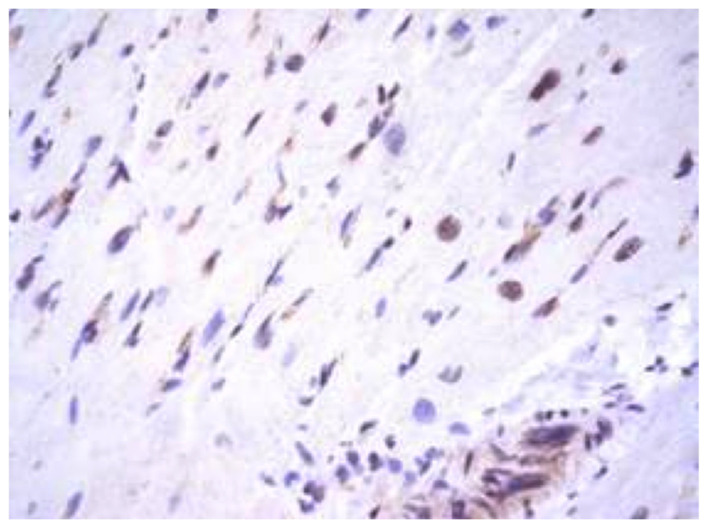
Expression of Epstein–Barr virus antigens (brown staining) in cardiomyocytes, lymphocytes, and endotheliocytes in generalized Epstein–Barr virus infection. IHH, mouse monoclonal anti-EBV (Thermo, Waltham, MA, USA), DAB. SW. ×200.

The Epstein–Barr virus (EBV) may result in infectious mononucleosis coupled to markedly affected lymphoid tissue and parenchymal organs [49]. It may persist throughout human life and cause severe chronic active infection of myocardial CD8+ T cells during ongoing perimyocarditis [50]. During myocarditis, expression of EBV antigens is detected in cardiomyocytes, lymphocytes, and vascular endothelium (Figure 4).

Enteroviruses (the Picornaviridae family) comprise a group of pathogens causing infectious diseases characterized by seasonality, high contagiousness, undulating course, and a polymorphic clinical picture. Enteroviruses infect cardiomyocytes by binding to a common transmembrane receptor (the Coxsackie and adenovirus receptor (CAR)), thereby resulting in direct myocardial damage, including destruction of their cytoskeleton, and triggering uncontrolled immune response even after virus elimination [51].

These viruses represent cytolytic viruses, causing myocarditis by inducing viral replication within host cells, followed by lysis and virus release. Most often, enteroviruses persist in myocytes, cardiomyocytes, and lymphoid tissue [52]. Currently, 87 enterovirus serotypes have been identified, 64 of which are pathogenic to humans. It was uncovered that high prevalence of Coxsackie viruses—mainly by serotypes A9, A13, A18, B3, and B4—was observed in subjects with myocarditis and cardiomyopathies.

**Figure 5:**
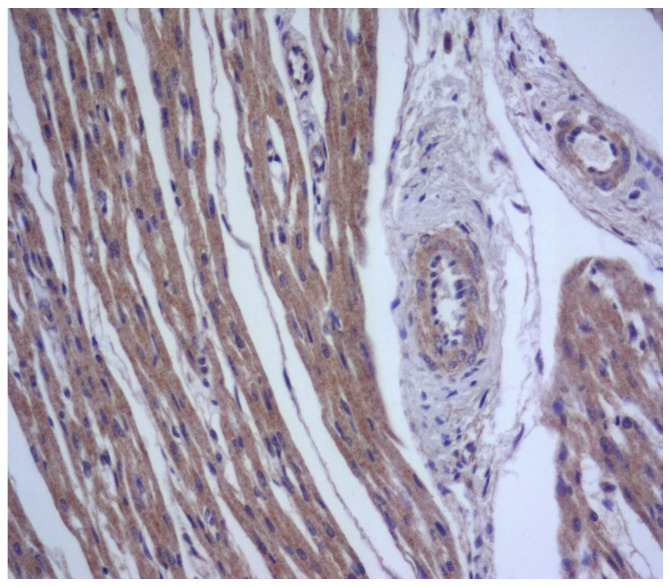
Expression of enterovirus antigens (brown staining) in cardiomyocytes, smooth muscle cells of blood vessels, and lymphocytes in enterovirus myocarditis. IHH, mouse monoclonal anti-Enterovirus VP-1-specific staining (Leica, New York, NY, USA), DAB. SW. ×200.

Importantly, infection with Coxsackie viruses upon myocarditis and dilated cardiomyopathy ranges from 73% to 82% and 38 to 75% of cases, respectively [53]. The expression of the virus-related VP-1 antigen in myocarditis is found in the cytoplasm of cardiomyocytes, endotheliocytes, vascular smooth muscle cells, fibroblasts, and inflammatory infiltrate cells (Figure 5).

**Figure 6:**
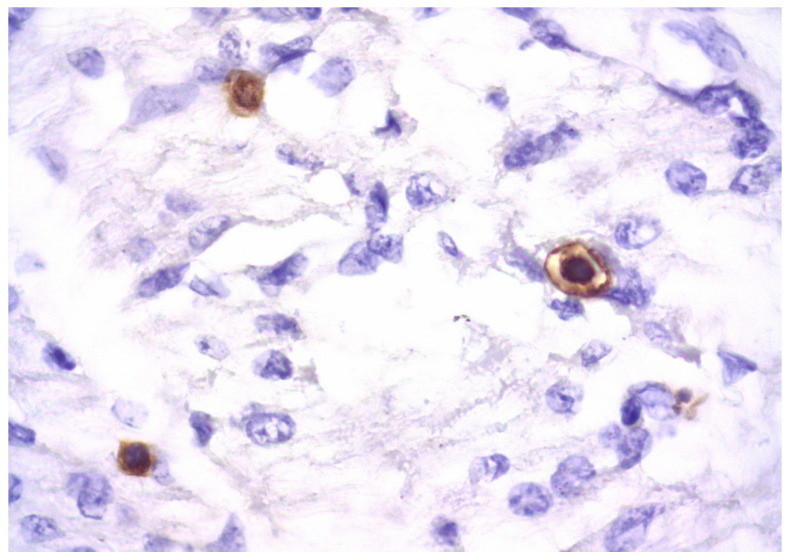
Expression of Parvovirus B19 antigens (brown staining) in cardiomyocytes. IHH, mouse monoclonal anti-Parvovirus B-19-specific staining (Leica, New York, NY, USA). SW. ×1000.

Parvovirus B19, of the Parvoviridae family, causes autoimmune reactions, acting as a trigger for autoimmune connective tissue diseases, wherein an autoimmune cross-reaction with autoantibodies against type II collagen, keratin, thyroglobulin, and cardiolipin has been verified. Parvovirus infections proceed usually in a mild course, but can also cause septic and hematological complications. Parvovirus B19 can result in developing myocarditis, vasculitis, pneumonia, fulminant hepatitis, and meningoencephalitis [54,55]. Interestingly, virus antigens in myocarditis are expressed on the sarcoplasmic membrane and nuclear membrane, as well as nuclei of cardiomyocytes, vascular endothelium, and inside inflammatory infiltrate cells [56]. At the same time, the virus-induced transformation of the affected cells renders them a “lantern” appearance (Figure 6).

Parvovirus-caused diffuse and focal myocarditis are characterized by focal hemorrhages and small focal myocardial infarctions due to endothelial damage, followed by necrosis of the vascular wall and thrombosis [57]. More often, focal myocarditis is detected in the subepicardial regions of the left ventricular posterolateral wall in magnetic resonance imaging. In addition, parvovirus may also trigger pericarditis and polyserositis [58], being often found in the pericardial fluid. The parvovirus B19 NS1 protein is considered to be the major protein, playing a crucial role in myocarditis pathogenesis [59]. Parvovirus B19 can enter endothelial cells and trigger toxic NS1-mediated release of pro-inflammatory cytokines by activating viral replication, expression of interleukin 6, tumor necrosis factor-α, initiating apoptosis via activated caspases 3 and 9, whereas viral protein VP-1 acts via cytotoxic T-cell activation. Prolonged severe cardiac inflammation, especially concomitant with acute B19V infection, may result in cardiomyocyte necrosis [60,61].

The etiological role of parvovirus infection in developing myocarditis has been continuously debated because this virus is often found in cardiac tissue of myocardium-lacking individuals. It can be assumed that parvovirus B19 DNA detected in the myocardium may suggest that it is a nonspecific bystander rather than a major causative agent of myocarditis [61].

Thus, we compared the morphological changes identified after infection with diverse viral agents (Table 1).

Thus, no specific unambiguous morphological criteria have been currently described for diagnosis of SARS-CoV-2-associated myocarditis. It should be noted that SARS-CoV-2 infection largely elicits a set of detected histopathological signs such as cardiomyocyte degeneration, apoptosis and necrosis, moderate interstitial hyperemia, myocardial tissue oedema, and capillary endothelial cell dysfunction [9,10].

Another clinicopathological subtype of this disease presents as acute myocarditis [62], usually being manifested by systolic ventricular dysfunction with or without ventricular dilatation [63]. The clinical picture of acute myocarditis may also vary from minor symptoms to cardiovascular manifestations with a high risk of sudden cardiac death, development of severe heart failure, refractory arrhythmias, and cardiogenic shock [63,64].

Clearly, the emergence of a new viral infection may require performance of differential diagnostics, to distinguish between myocarditis associated with SARS-CoV-2 and other viral agents. A comparative analysis is presented in Table 2.

Pediatric myocarditis associated with COVID-19 is manifested by tachycardia (up to 17%) and a high incidence of hypotension (up to 63%) (Table 2). In contrast, myocarditis of other origin was found to be characterized by clinical symptoms with a higher rate of arrhythmia (45%), tachycardia (57%), and tachypnoea (52%).

PCR-based SARS-CoV-2 RNA detection represents the only evidence-based criterion for diagnosis of related myocarditis. Similar issues are also observed while assessing myocardial lesions in other viral infections. Myocarditis-related etiological diagnostics may be provided while comprehensively assessing diagnostic criteria obtained, verifying virus exposure, followed by developing overt clinical symptoms complimented with MRI and CT changes.

## 4. Discussion

Myocarditis is one of the leading causes of dilated cardiomyopathy (27%) and heart transplantation (80%) in children lacking congenital heart defects [73]. Upon spontaneous recovery, inflammatory alterations elicit irreversible damage to the myocardium and subsequent severe systemic complications [65,74,75]. The initial diagnosis of myocarditis is usually based on the clinical presentation, which may be complicated due to the wide range and variety among associated symptoms [62,66]. Depending on the severity of COVID-19, myocarditis has been reported to occur in 17% to 53% of cases [9,10].

Currently, several types of clinical forms of myocarditis have been described, among which, the most threatening is fulminant myocarditis, described as a distinct subgroup of acute myocarditis [62,64,67].

This course of myocarditis is characterized by an acute and sudden onset after prominent manifestations of viral prodrome, severe heart diffuse inflammation, multiple foci of active myocarditis based on histology examination, rapid development of hemodynamic collapse, ventricular dysfunction (which is able to result in the patient’s death due to cardiogenic shock), fetal ventricular tachyarrhythmias or bradyarrhythmias, and multiple organ failure [63,64]. In this case, the cavity of the left ventricle has a normal size, coupled to septal thickening [68]. All patients with fulminant myocarditis require inotropic therapy or mechanical circulatory support to maintain tissue perfusion until heart transplantation or recovery [17,73,74]. The clinical manifestations of this course of myocarditis vary widely, with or without systemic manifestations of infection or inflammatory disease. The most common symptoms are shortness of breath accompanied by chest pain and arrhythmias such as atrial fibrillation, ventricular tachycardia, or heart block, leading to sudden cardiac death [74,75].

At the same time, as noted earlier, the incidence of myocarditis is probably underestimated by taking into account the non-specific nature of the symptoms, often reminiscent of more common conditions such as bronchial asthma or viral gastroenteritism as well as a wide spectrum of the disease-related clinical signs [76,77]. Comprehensive clinical evaluation is critical in the diagnosis of myocarditis because it assigns the disease to the category of clinically suspected myocarditis diseases, based on additional decisions necessary for improved diagnostics and therapy.

As noted earlier, myocarditis remains a rather high-priority issue in pediatric cardiology, mainly due to the difficulty in detecting and diagnosing this disease [76,77]. Hence, the methods for defining myocarditis, as well as the criteria for diagnosing it, are being revised annually.

Initially, the diagnosis of myocarditis should be based on pathologic evidence of inflammatory cardiomyopathy, but the frequency of diagnostic biopsies has now declined because, in diagnostic strategy, practitioners have been increasingly relying on magnetic resonance imaging (MRI) as well as clinical and laboratory criteria [62,78]. The paradigm shift in the diagnosis of myocarditis coincides with the advances in MRI, but requires further investigation of its accuracy in this context. A positive biopsy confirms detected myocarditis; however, regarding MRI, a negative result does not necessarily exclude this pathology. This is also true for pediatric COVID-19-associated myocarditis [9,79]. However, even in SARS-CoV-2-related myocarditis, validation of MRI parameters in children is necessary, to determine the timing and criteria for diagnostics of pediatric acute myocarditis in COVID-19 [78].

Clinically suspected myocarditis includes a combination of anamnestic and symptomatic signs, confirming the disease in cases where an MRI or biopsy cannot be performed, despite the negative data they provide [20,28,78].

To date, no definitive criteria have been developed that can be used to confirm myocarditis solely by clinical signs or to differentiate clinical suspicion from potential myocarditis.

It has been recognized that myocarditis, both in adults and children, can mimic myocardial infarction with severe chest pain and typical ECG changes, along with increased serum creatinine kinase level and normal coronary angiograms [62]. In this regard, it is important that parvovirus B19 as well as adenovirus and Epstein–Barr virus are found in the myocardium of such patients [80].

Studies of sudden cardiac death syndrome in the pediatric population have linked viral infections due to enterovirus, adenovirus, parvovirus B19, and Epstein–Barr virus as well as myocarditis with victims of sudden infant death syndrome [57,60,75,81]. In the United States, myocarditis accounts for 9% of sudden death cases of young athletes with a documented CV event [79].

SARS-CoV-2 causes damage to vascular endothelial cells and impairs their normal functioning, which further induces increased blood clotting and clot formation, ultimately leading to emerging cardiac abnormalities [77,82].

The detection of late gadolinium enhancement (LGE), characterizing myocyte necrosis, has been described in a small percentage of patients with COVID-19 vs. non-COVID-19 myocarditis. This may suggest a different pathogenetic pattern of heart damage related to SARS-CoV-2 [13]. The presence of the detected changes may be accompanied by very scant clinical manifestations, wherein a decision to perform MRI may be forced only if a child’s condition deteriorates [9,10,13,82].

## 5. Conclusions

Morphological criteria used for diagnostics of SARS-CoV-2-caused myocarditis require further revision. PCR-based SARS-CoV-2 RNA detection represents the only evidence-based criterion for diagnosis of related myocarditis. Similar issues arise while diagnosing myocardial lesions caused by other viral infections. Evidence for an etiological diagnosis of myocarditis may be provided by comprehensively analyzed diagnostic criteria, confirming virus exposure along with developing specific clinical symptoms, MRI and CT changes, and morphological criteria.

## Figures and Tables

**Table 1:** Morphological alterations associated with SARS-CoV-2 and other viral infections.

Etiologic Agent	Morphological Alterations
SARS-CoV-2	Focal polymorphocytic myocarditis. Degeneration, apoptosis, cardiomyocyte necrosis, moderate interstitial hyperemia, myocardial tissue oedema.
Herpes simplex viruses	Focal or diffuse lymphocytic or polymorphocytic myocarditis, with rare necrotic leucocytic myocarditis. Necrosis with dusty or globular nuclear rexis of dead cells and scanty perifocal infiltration with polymorphonuclear leukocytes.
Cytomegalovirus	Focal or diffuse lymphocytic or polymorphocytic myocarditis, with rare necrotic leucocytic myocarditis. Virus-induced alteration of vascular endothelial cells. Cell infection not always accompanied by gigantocellular transformation, may result in morphological hypodiagnostics of organ lesions.
Herpes virus type 6	Focal polymorphocytic myocarditis, more often in the basal interventricular septum
The Epstein-Barr virus	Focal or diffuse lymphocytic or polymorphocytic myocarditis. Cytopathic changes in vascular endothelial cells, cardiomyocytes
Enteroviruses Coxsackie viruses A9, A13, A18, B3, and B4	Focal or diffuse lymphocytic or polymorphocytic myocarditis. Direct viral damage to the myocardium, including destruction of related cytoskeleton. Persistence of viruses in cardiomyocytes, blood vessel smooth muscle cells, and lymphocytes
Parvovirus B19	Focal or diffuse lymphocytic or polymorphocytic myocarditis. Virus-induced transformation of parenchymatous and non-parenchymatous cells. Extensive hemorrhage and non-coronary myocardial necrosis due to endothelial damage followed by thrombosis.

**Table 2:** Comparative characteristics of myocarditis of different etiologies.

Complaints and Symptoms	Non-SARS-CoV-2 Myocarditis	SARS-CoV-2 Myocarditis
Alterations in the Cardiovascular System		
Arrhythmias (ventricular and atrial)	45% [23,50,62,63]	No data
Tachycardia	57% [65,66,67,68]	8.9–17.9% [69,70]
Tachypnea	52% [1,2,15]	No data
Heart murmur, systolic hypotension	20–25% [1,3]	32–63% [71,72]

## Data Availability

Not applicable.
